# Long-Term Exposure to Fine Particulate Matter: Association with Nonaccidental and Cardiovascular Mortality in the Agricultural Health Study Cohort

**DOI:** 10.1289/ehp.1307277

**Published:** 2014-03-14

**Authors:** Scott Weichenthal, Paul J. Villeneuve, Richard T. Burnett, Aaron van Donkelaar, Randall V. Martin, Rena R. Jones, Curt T. DellaValle, Dale P. Sandler, Mary H. Ward, Jane A. Hoppin

**Affiliations:** 1 Health Canada, Air Health Sciences Division, Ottawa, Ontario, Canada; 2 Department of Occupational and Environmental Health, University of Montreal, Montreal, Quebec, Canada; 3 Health Canada, Population Studies Division, Ottawa, Ontario, Canada; 4 Institute of Health: Science, Technology and Policy, Carleton University, Ottawa, Ontario, Canada; 5 Department of Physics and Atmospheric Science, Dalhousie University, Halifax, Nova Scotia, Canada; 6 Harvard-Smithsonian Center for Astrophysics, Cambridge, Massachusetts, USA; 7 Occupational and Environmental Epidemiology Branch, Division of Cancer Epidemiology and Genetics, National Cancer Institute, National Institutes of Health, Department of Health and Human Services, Bethesda, Maryland, USA; 8 Epidemiology Branch, National Institute of Environmental Health Sciences, National Institutes of Health, Department of Health and Human Services, Research Triangle Park, North Carolina, USA

## Abstract

Background: Few studies have examined the relationship between long-term exposure to ambient fine particulate matter (PM_2.5_) and nonaccidental mortality in rural populations.

Objective: We examined the relationship between PM_2.5_ and nonaccidental and cardiovascular mortality in the U.S. Agricultural Health Study cohort.

Methods: The cohort (*n* = 83,378) included farmers, their spouses, and commercial pesticide applicators residing primarily in Iowa and North Carolina. Deaths occurring between enrollment (1993–1997) and 30 December 2009 were identified by record linkage. Six-year average (2001–2006) remote-sensing derived estimates of PM_2.5_ were assigned to participants’ residences at enrollment, and Cox proportional hazards models were used to estimate hazard ratios (HR) in relation to a 10-μg/m^3^ increase in PM_2.5_ adjusted for individual-level covariates.

Results: In total, 5,931 nonaccidental and 1,967 cardiovascular deaths occurred over a median follow-up time of 13.9 years. PM_2.5_ was not associated with nonaccidental mortality in the cohort as a whole (HR = 0.95; 95% CI: 0.76, 1.20), but consistent inverse relationships were observed among women. Positive associations were observed between ambient PM_2.5_ and cardiovascular mortality among men, and these associations were strongest among men who did not move from their enrollment address (HR = 1.63; 95% 0.94, 2.84). In particular, cardiovascular mortality risk in men was significantly increased when analyses were limited to nonmoving participants with the most precise exposure geocoding (HR = 1.87; 95% CI: 1.04, 3.36).

Conclusions: Rural PM_2.5_ may be associated with cardiovascular mortality in men; however, similar associations were not observed among women. Further evaluation is required to explore these sex differences.

Citation: Weichenthal S, Villeneuve PJ, Burnett RT, van Donkelaar A, Martin RV, Jones RR, DellaValle CT, Sandler DP, Ward MH, Hoppin JA. 2014. Long-term exposure to fine particulate matter: association with nonaccidental and cardiovascular mortality in the Agricultural Health Study Cohort. Environ Health Perspect 122:609–615; http://dx.doi.org/10.1289/ehp.1307277

## Introduction

Numerous cohort studies have examined the relationship between long-term exposures to ambient fine particulate matter (≤ 2.5 μm; PM_2.5_) and nonaccidental and cardiovascular mortality (Beelen et al. 2008; Cesaroni et al. 2013; Chen et al. 2005; Crouse et al. 2012; Dockery et al. 1993; Katanoda et al. 2011; Hoek et al. 2013; Laden et al. 2006; Lepeule et al. 2012; Miller et al. 2007; Ostro et al. 2010; Pope et al. 2002, 2004; Puett et al. 2009, 2011). In general, findings from these studies support a causal relationship between chronic PM_2.5_ exposure and mortality, likely owing to biological mechanisms including altered autonomic function, impaired vascular function, and increased pulmonary and systemic inflammation (Brook et al. 2010). However, existing evidence primarily reflects associations that have been observed in urban areas, where air monitoring networks are located. To date, few studies have explored the potential relationship between long-term exposure to PM_2.5_ and mortality in rural areas; however, some evidence suggests that the association between PM_2.5_ and life expectancy may be stronger in more urban, densely populated regions (Correia et al. 2013). Indeed, because PM_2.5_ is a heterogeneous mixture, it is possible that the long-term health impacts of ambient PM_2.5_ may differ between urban and rural areas owing to differences in particle composition. The emergence of remote sensing–based estimates of ground-level PM_2.5_ (van Donkelaar et al. 2010) now makes it possible to include rural areas in large-scale air pollution studies, as was recently demonstrated in a Canadian national-level cohort study (Crouse et al. 2012). Moreover, recent evidence suggests that studies in rural areas are relevant, given that low concentrations (< 10 μg/m^3^) of ambient PM_2.5_ have been associated with increased nonaccidental and cardiovascular mortality (Crouse et al. 2012).

Here we used remote sensing methods to evaluate the relationship between long-term exposure to PM_2.5_ and nonaccidental and cardiovascular mortality in the Agricultural Health Study cohort (Alavanja et al. 1996). Two features of this cohort are important for studying associations between ambient PM_2.5_ and mortality: First, by virtue of their occupations, private applicators (i.e., farmers) in the cohort are more likely to spend a greater proportion of their time working outdoors close to home relative to the general population. Therefore, exposure misclassification should be reduced relative to other cohort studies that have assigned exposures to place of residence. Second, individual-level data were collected that allowed us to control for potentially important confounding factors (e.g., smoking) and to classify individuals based on the estimated amount of time they typically spent outdoors.

## Methods

*Study population*. The Agricultural Health Study (AHS) was designed to evaluate the potential health effects of agricultural exposures among commercial pesticide applicators, farmers, and their families in Iowa and North Carolina, USA. Details of the AHS design have been described (Alavanja et al. 1996). Briefly, participants were recruited from pesticide-licensing facilities between 1993 and 1997 and enrolled by completing a self-administered questionnaire. Applicators who completed the enrollment questionnaire were also asked to complete a more detailed take-home questionnaire and were provided with a questionnaire to be completed by their spouse. In total, 82% of farmers, 47% of commercial applicators, and 75% of spouses eligible to participate in the study were enrolled. Of these, 52,987 applicators (93%) (48,074 private applicators and 4,913 commercial applicators) and 30,391 spouses (94%) were included in the present analysis because geographic coordinates were available for these participants. Most participants lived in either Iowa or North Carolina, but a small fraction of participants (~ 1%) lived outside these states. All individual-level covariate data used in the analyses were collected from the enrollment questionnaire, the take-home questionnaire, or the spouse questionnaire completed at the time of enrollment. The study was approved by the institutional review boards of the U.S. National Institutes of Health, its contractors, and by Health Canada’s Research Ethics Board. Informed consent was implied by the return of study questionnaires, as approved by institutional review boards.

*Outcome classification*. Deaths were identified through annual linkage with death registries in Iowa and North Carolina as well as the National Death Index. Underlying causes of death were coded using the *International Classification of Diseases* (ICD) in effect at the time of death (9th or 10th Revision) (World Health Organization 1977, 1992). Analysis of nonaccidental mortality included ICD-9 codes lower than 800 and ICD-10 codes lower than V01. Analysis of cardiovascular mortality included ICD-9 codes 400–440 and ICD-10 codes I10–I70. As an exploratory analysis, we also examined several specific causes of death including ischemic heart disease (ICD-10 code: I25), cerebrovascular disease (ICD-10 codes: I60–I69), and lung cancer (ICD-10 code: C34).

*PM_2.5_ exposure assignment*. Agricultural Health Study addresses were batch matched using automated geocoding methods (ArcGIS Geocoding Engine version 2; ESRI, Redlands, CA, USA) to obtain the latitude and longitude (30-foot street offset) at the enrollment residence. Additionally, Iowa addresses that were not matched or that were matched to a ZIP code, town name, or street name only were manually reviewed, corrected if necessary, and entered into ArcGIS or Google Maps and other online mapping software to obtain coordinates. Additional address cleaning was conducted in Iowa as part of a separate project and has not been completed for North Carolina. In total, approximately 82% of participants were successfully matched to a specific street address, 2% were matched to a street name (at the center-point of the street segment if the street was located within one ZIP code), and 15% were matched to ZIP code centroids. A small number of participants (*n* = 62, 0.1%) were geocoded to a city centroid without further spatial resolution.

Satellite-based estimates of ambient PM_2.5_ concentrations were assigned to each enrollment address with a spatial resolution of approximately 10 × 10 km. Specifically, satellite retrievals of the total atmospheric column at each address were related to surface concentrations using column-to-surface ratios from the GEOS-Chem chemical transport model (GEOS-Chem 2012). PM_2.5_ concentrations assigned to each location reflected the 6-year mean (2001–2006) of combined daily aerosol optical depth retrievals from the MODIS (Moderate Resolution Imaging Spectroradiometer) (Levy et al. 2007) and MISR (Multi-angle Imaging SpectroRadiometer) (Diner et al. 2005) instruments and were corrected for sampling bias as previously described (van Donkelaar et al. 2010). The 2001–2006 satellite estimates were based on an average of 780 values (per 10 × 10 km grid) for North Carolina and 510 values for Iowa. The impact of missing values was reduced by applying the coincidently to noncoincidently sampled ratio from the GEOS-Chem chemical transport model; this approach corrects for bias that may result from days lost due to the presence of clouds or snow (van Donkelaar et al. 2010).

Although this time period does not cover the entire duration of follow-up, existing ground-level data from the IMPROVE (Interagency Monitoring of Protected Visual Environments) network suggests that the years 2001–2006 may be generally representative of annual average ambient concentrations between 1995 and 2009. For example, one site (SHRO1) in North Carolina collected PM_2.5_ data from 1995 forward, and the mean PM_2.5_ concentration at this site was 6.4 μg/m^3^ between 1995 and 2009, whereas the 2001–2006 average was 5.8 μg/m^3^ and the 2001–2009 average was 5.4 μg/m^3^. Unfortunately, additional ground-level monitoring data are not available to conduct similar comparisons at multiple sites over the entire follow-up period (1993–2009); however, these data generally support the use of a 6-year average (2001–2006) to estimate long-term exposures to ambient PM_2.5_ in the AHS cohort. In general, previous studies have reported strong correlations between satellite-based estimates of ambient PM_2.5_ and ground-level measurements in both Canada (*r* = 0.84) (Crouse et al. 2012) and North America as a whole (*r* = 0.77) (van Donkelaar et al. 2010). Correlations between satellite and ground-level data are slightly lower for Iowa (*r* = 0.63; 34 sites) and North Carolina (*r* = 0.54; 63 sites) specifically (see Supplemental Material, Figure S1).

Residential address information collected at enrollment was updated through annual mailings and during follow-up interviews in phase 2 (1999–2005) and phase 3 (2005–2010) of the AHS. Participants were classified as nonmovers if their residential address did not change over time. In addition to moving, address changes could occur if a rural route was updated to a street address; however, these participants were not included among nonmovers. Detailed time-varying address/exposure data were not available for this analysis, but we were able to verify which participants remained at their enrollment address (i.e., nonmovers). A small number of participants (*n* = 229) provided multiple enrollment addresses (up to 3). For these participants, the address with the lowest PM_2.5_ value was used in the analysis; however, PM_2.5_ values at multiple addresses typically varied by < 1–2 μg/m^3^.

*Statistical analysis*. Hazard ratios (HR) and their 95% CIs were estimated using the Cox proportional hazards model with AHS data release AHSREL201103.00 (unpublished data). Survival times were calculated from the age at enrollment to age at death or the end of follow-up (30 December 2009). Age in years was used as the time axis, and all models were adjusted for sex, state of enrollment (Iowa/North Carolina), and birth year category (< 1930, 1930–1939, 1940–1949, 1950–1959, ≥ 1960). Race was not included as a stratifying variable or as a covariate in statistical models because participants were predominantly Caucasian (> 98%).

We examined three separate models to evaluate the relationship between ambient PM_2.5_ and nonaccidental and cardiovascular mortality. Potential confounding factors included in these models were selected based on previous studies of long-term exposure to PM_2.5_ and mortality. Simple models (including only PM_2.5_) were examined first, followed by models including potentially important behavioral/personal factors (e.g., smoking), and finally fully adjusted models including socioeconomic factors and additional lifestyle factors (e.g., education, diet). Specifically, minimally adjusted models included PM_2.5_ as a continuous variable and the factors mentioned above. Moderately adjusted models included additional terms for continuous measures of both body mass index (BMI; kilograms per meter squared) and pack-years of smoking. Fully adjusted models included additional indicator variables for marital status (married or living as married, divorced or separated, widowed, never married), education (1–8 years, some high school, high school graduate, high school equivalency, 1–3 years vocational education beyond high school, some college, college graduate, some graduate school, other), alcohol consumption at enrollment (< 1, 1–5, ≥ 6 drinks per month), and vegetable servings per week (< 3, 3–4, 5–6, ≥ 7). Categories for missing values were included as part of indicator variables for alcohol consumption and vegetable servings per week to minimize reductions in sample size owing to missing values. Models including both squared and linear terms for BMI and pack-years of smoking were also evaluated, but squared terms were not included in final models because they did not appreciably change the coefficients for PM_2.5_ or improve model fit based on the Akaike Information Criterion (AIC).

Additional analyses were conducted among nonmovers (as defined above) as well as models stratified by the estimated amount of time spent outdoors. Time outdoors was assessed through the following question: “In the growing season, how many hours a day do you generally spend in the sun?” The median value of this response was used as the cut point in stratified analyses (this value differed for men and women). Formal tests for interactions between ambient PM_2.5_ and sex, state of enrollment, BMI, and time spent outdoors were conducted by evaluating the statistical significance of the corresponding first-order interaction terms. Finally, concentration–response functions were graphed using natural splines for PM_2.5_ with two degrees of freedom using adjusted Cox survival models. Natural splines with 3 and 4 degrees of freedom were explored but did not improve model fit (data not shown). A *p*-value of 0.05 was used to indicate statistical significance.

We conducted sensitivity analyses to evaluate whether occupational sources of PM_2.5_ exposure and/or additional smoking variables may influence the findings. For occupational exposures, sensitivity analyses evaluated indicator variables for activities including repairing engines, grinding metal, welding, and driving diesel tractors as well as continuous variables for both years of mixing or applying pesticides and physical activity (hours exercising/week). Continuous measures of years mixing or applying pesticides were moderately correlated with ever/never use of specific pesticides classes including herbicides, phenoxy herbicides, insecticides, fungicides, carbamates, organophosphates, and organochlorines (0.49 < Spearman’s *r* < 0.65) and thus these factors were not examined individually. Ambient PM_2.5_ concentrations were not correlated with ever/never use of any of these broad pesticide use categories (*r* < 0.21). Because data for several of these factors were limited to a subset (~ 40% of eligible applicators) of the cohort (Tarone et al. 1997), the primary purpose of this sensitivity analysis was to evaluate the potential impact of omitting these factors on effect estimates for ambient PM_2.5_. Similarly, additional smoking variables were evaluated in combination with pack-years of smoking including continuous measures of cigarettes per day and years smoked as well as indicator variables for smoking status at enrollment (current, former, never) and ever/never use of pipes, cigars, or chewing tobacco.

We examined models replacing missing BMI and vegetable intake data with sex-specific mean values to evaluate the potential impact of missing data on observed associations. In addition, models for cardiovascular mortality were stratified by BMI (above/below median value) to evaluate potential effect modification by BMI. Furthermore, models were examined excluding participants with exposures assigned to ZIP code centroids to evaluate the potential impact of exposure measurement error resulting from geocoding error. Finally, models were examined including a random effect (i.e., frailty term) for county to evaluate the potential impact of spatial correlations on the PM_2.5_ coefficients. These models were examined separately for men and women; however, owing to sample size limitations, this analysis was limited to minimally adjusted models and nonmovers were not examined separately.

All HRs reflect a 10-μg/m^3^ increase in ambient PM_2.5_ concentrations, and all statistical analyses were conducted using STATA version 11 (StataCorp, College Station, TX, USA). Concentration–response plots for PM_2.5_ were generated in R (version 2.15.2; http://www.r-project.org/foundation/).

## Results

**Figure 1:**
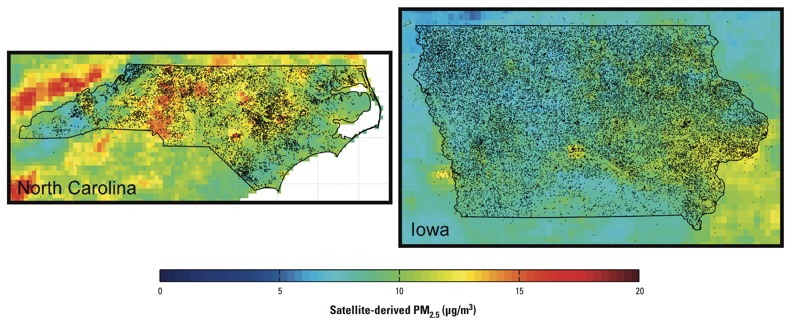
Spatial distribution of participants and estimated PM_2.5_ concentrations (μg/m^3^) in North Carolina (left) and Iowa (right). Dots outside state lines reflect the small number of participants who were enrolled in the study but lived outside North Carolina or Iowa.

Participant characteristics at the time of enrollment are presented in Table 1. Participants were predominantly male (62%), private applicators (i.e., farmers) (58%), residents of Iowa (70%), and never-smokers (58%). On average, men and women were 46 and 47 years of age at enrollment, respectively; men reported a median value of 6–10 hr/day in the sun (i.e., outdoors) during the growing season, whereas women reported a median value of 1–2 hr/day. Ambient PM_2.5_ concentrations were slightly higher for participants enrolled in North Carolina than in Iowa (mean difference = 2.27 μg/m^3^; 95% CI: 2.25, 2.29) with estimated mean concentrations ranging between 5.7 and 19.2 μg/m^3^. Nearly all PM_2.5_ (99%) values in Iowa were below the current annual National Ambient Air Quality Standard of 12 μg/m^3^ (U.S. Environmental Protection Agency 2012), whereas 69% of values were below this standard in North Carolina. The majority (61%) of participants did not have an address update from their enrollment address (i.e., were classified as nonmovers), and this proportion was similar among men (60%) and women (62%). On average, ambient PM_2.5_ concentrations were similar for nonmovers (mean, 9.58 μg/m^3^) relative to the cohort as a whole (mean, 9.44 μg/m^3^). The spatial distribution of participants and the estimated PM_2.5_ concentrations in Iowa and North Carolina are shown in Figure 1. In total, the analysis was based on nearly 1.2 million person-years of follow-up, with 5,931 nonaccidental deaths and 1,967 cardiovascular deaths. The median follow-up time was 13.9 years.

**Table 1:** AHS participant characteristics at enrollment (1993–1997).

Characteristic	*n* (%)	PM_2.5_ (μg/m^3^) (mean ± SD)
Overall	83,378 (100)	9.52 ± 1.66
Sex
Male	51,807 (62)	9.54 ± 1.66
Female	31,571 (38)	9.50 ± 1.65
Participant type
Private applicator	48,074 (58)	9.60 ± 1.69
Commercial applicator^*a*^	4,913 (6)	9.04 ± 1.28
Spouse	30,391 (36)	9.48 ± 1.64
Mobility
Nonmovers	50,590 (61)	9.58 ± 1.70
Age at enrollment (years)
< 40	27,745 (33)	9.40 ± 1.59
40–59	41,123 (49)	9.52 ± 1.66
60–79	14,271 (17)	9.77 ± 1.76
≥ 80	239 (1)	10.21 ± 1.72
State of enrollment
Iowa	58,113 (70)	8.84 ± 1.13
North Carolina	25,265 (30)	11.11 ± 1.61
Marital status
Married/living as married	74,461 (89)	9.51 ± 1.66
Divorced/separated	2,368 (3)	9.72 ± 1.67
Widowed	538 (< 1)	9.85 ± 1.76
Never married	5,828 (7)	9.54 ± 1.64
Missing	183 (< 1)	10.80 ± 1.83
Smoking status
Never	48,259 (58)	9.40 ± 1.60
Former	20,678 (25)	9.60 ± 1.71
Current	11,265 (14)	9.76 ± 1.74
Missing	3,176 (3)	10.03 ± 1.76
Smoking status (pack-years)	
0	48,548 (55)	9.40 ± 1.60
0.1–15	19,595 (22)	9.55 ± 1.68
> 15	15,235 (17)	9.88 ± 1.77
Missing	4,464 (6)	10.04 ± 1.76
BMI (kg/m^2^)
< 25	23,218 (28)	9.51 ± 1.66
25–30	28,267 (34)	9.47 ± 1.64
> 30	14,076 (17)	9.58 ± 1.67
Missing	17,817 (21)	9.62 ± 1.68
Alcohol consumption (drinks/month)
< 1	28,367 (34)	9.73 ± 1.74
1–5	25,535 (31)	9.21 ± 1.49
≥ 6	24,784 (30)	9.35 ± 1.57
Missing	4,692 (5)	10.07 ± 1.77
Highest level of schooling
High school or less	40,915 (49)	9.57 ± 1.66
Beyond high school	39,183 (47)	9.48 ± 1.66
Missing	3,280 (4)	10.05 ± 1.77
Vegetable servings per week	
< 3	6,782 (8)	9.43 ± 1.56
3–4	25,047 (30)	9.43 ± 1.62
5–6	19,777 (24)	9.25 ± 1.50
≥ 7	20,366 (24)	9.79 ± 1.78
Missing	11,406 (14)	9.80 ± 1.74
^***a***^Iowa only.		

HRs describing the relationships between ambient PM_2.5_ and nonaccidental and cardiovascular mortality are shown in Table 2. Ambient PM_2.5_ concentrations were not associated with an increased risk of nonaccidental mortality for the cohort as a whole; however, significant inverse relationships were observed among nonmoving women in moderately and fully adjusted models. Conversely, positive associations were observed between ambient PM_2.5_ and cardiovascular mortality in men for all models examined. In addition, the strength of this association increased when analyses were limited to men who did not move from their enrollment address, and tests for PM_2.5_–sex interactions for cardiovascular mortality were statistically significant in moderately (*p* = 0.05) and fully adjusted (*p* = 0.02) models among nonmovers.

**Table 2:** HRs for nonaccidental and cardiovascular mortality per 10‑μg/m^3^ increase in ambient PM_2.5_ among participants in the AHS, 1993–2011.

Cause of death	Minimally adjusted^*a*^		Moderately adjusted^*b*^		Fully adjusted^*c*^	
	No. of deaths	HR (95% CI)	No. of deaths	HR (95% CI)	No. of deaths	HR (95% CI)
Nonaccidental^*d*^
All	5,929	0.92 (0.76, 1.11)	4,069	0.94 (0.75, 1.18)	3,961	0.95 (0.76, 1.20)
Men	4,271	0.97 (0.78, 1.20)	2,795	1.00 (0.76, 1.31)	2,717	1.05 (0.80, 1.39)
Women	1,658	0.80 (0.56, 1.15)	1,274	0.82 (0.54, 1.24)	1,244	0.78 (0.51, 1.19)
Nonmovers
All	4,019	0.85 (0.68, 1.06)	2,710	0.81 (0.61, 1.07)	2,631	0.85 (0.64, 1.13)
Men	2,860	0.93 (0.71, 1.21)	1,830	0.95 (0.68, 1.32)	1,772	1.03 (0.73, 1.44)
Women	1,159	0.67 (0.44, 1.03)	880	0.57 (0.34, 0.95)	859	0.56 (0.33, 0.94)
Cardiovascular^*e*^
All	1,967	1.00 (0.72, 1.37)	1,305	1.15 (0.77, 1.71)	1,273	1.15 (0.76, 1.72)
Men	1,534	1.08 (0.75, 1.56)	973	1.37 (0.87, 2.17)	950	1.43 (0.89, 2.27)
Women	433	0.73 (0.37, 1.46)	332	0.67 (0.30, 1.51)	323	0.60 (0.26, 1.37)
Nonmovers
All	1,357	1.00 (0.67, 1.46)	905	1.13 (0.70, 1.82)	883	1.18 (0.73, 1.92)
Men	1,043	1.16 (0.75, 1.80)	670	1.51 (0.88, 2.60)	653	1.63 (0.94, 2.84)
Women	314	0.59 (0.26, 1.32)	235	0.46 (0.17, 1.23)	230	0.43 (0.16, 1.15)
Cardiovascular^*e*^–most precise exposure^*f*^
All	1,575	1.08 (0.75, 1.55)	1,079	1.25 (0.81, 1.94)	1,055	1.31 (0.84, 2.04)
Men	1,223	1.16 (0.77, 1.74)	805	1.52 (0.92, 2.51)	786	1.66 (1.00, 2.78)
Women	352	0.84 (0.39, 1.82)	274	0.68 (0.28, 1.67)	269	0.62 (0.25, 1.55)
Nonmovers
All	1,196	1.13 (0.75, 1.71)	818	1.22 (0.74, 2.03)	801	1.33 (0.80, 2.23)
Men	919	1.31 (0.82, 2.09)	606	1.67 (0.94, 2.96)	592	1.87 (1.04, 3.36)
Women	277	0.68 (0.28, 1.64)	212	0.46 (0.16, 1.30)	209	0.45 (0.16, 1.29)
^***a***^Age as follow-up time and adjusted for sex, state of enrollment, and birth year.^***b***^Minimally adjusted plus covariates for pack-years of smoking and BMI. ^***c***^Moderately adjusted plus covariates for marital status, education level, alcoholic drinks per month, and vegetable intake. ^***d***^ICD-10 codes < V01. ^***e***^ICD-10 codes I10–I70. ^***f***^Excluding participants with PM_2.5_ ­exposure assigned to a ZIP code centroid.						

Sensitivity analyses excluding participants with PM_2.5_ concentrations assigned to ZIP code centroids resulted in stronger relationships between ambient PM_2.5_ and cardiovascular mortality among men (Table 2). In particular, significant positive associations were observed between ambient PM_2.5_ and cardiovascular mortality in fully adjusted models for all men (HR = 1.66; 95% CI: 1.00, 2.78) and among male nonmovers (HR = 1.87; 95% CI: 1.04, 3.36). Changes were less dramatic for nonaccidental mortality; however, ambient PM_2.5_ was not associated with a significant decrease in nonaccidental mortality among women when we excluded participants with exposures assigned to ZIP code centroids (see Supplemental Material, Table S1).

There were no statistically significant interactions between PM_2.5_ and state of enrollment on the risk of cardiovascular or nonaccidental mortality (*p* > 0.05) (data not shown). Because state of enrollment was correlated with ambient PM_2.5_ (*r* = 0.60) we also examined models excluding this variable from the analysis. In these models, ambient PM_2.5_ was positively associated with cardiovascular mortality among men in minimally (HR = 1.32; 95% CI: 1.00, 1.76), moderately (HR = 1.41; 95% CI: 1.00, 2.02), and fully adjusted models (HR = 1.39; 95% CI: 0.96, 2.01). However, state of enrollment was associated with cardiovascular mortality and was moderately correlated with ambient PM_2.5_; therefore, we retained state of enrollment as a possible confounder in subsequent models despite potential underestimation of PM_2.5_ effects.

Of the specific causes of death examined, ischemic heart disease (*n* = 213) (HR = 2.68; 95% CI: 1.04, 6.87) and cerebrovascular mortality (*n* = 242) (HR = 1.78; 95% CI: 0.72, 4.42) were each positively associated with ambient PM_2.5_ in fully adjusted models, but lung cancer mortality (*n* = 337) was not positively associated with ambient PM_2.5_ (HR = 0.75; 95% CI: 0.34, 1.65).

When analyses were stratified by the estimated amount of time participants spent outdoors at enrollment (< 6 vs. ≥ 6 hr/day), associations between PM_2.5_ and cardiovascular mortality tended to be stronger among men who reported spending the most time outdoors (Table 3). However, small numbers of cardiovascular deaths were classified in each category of time spent outdoors, and formal tests of interaction between PM_2.5_ and time spent outdoors were not statistically significant (*p* > 0.05). There were too few deaths among women to support similar analysis among female participants.

**Table 3:** HRs for cardiovascular mortality among men per 10‑μg/m^3^ increase in ambient PM_2.5_ stratified by estimated time spent outdoors and BMI at enrollment.

Cardiovascular death^*a*^	Minimally adjusted^*b*^		Moderately adjusted^*c*^		Fully adjusted^*d*^	
	No. of deaths	HR (95% CI)	No. of deaths	HR (95% CI)	No. of deaths	HR (95% CI)
Time outdoors
All men
≥ 6 hr/day	487	1.32 (0.68, 2.56)	426	1.04 (0.52, 2.12)	423	1.08 (0.52, 2.22)
< 6 hr/day	289	1.19 (0.54, 2.66)	254	1.05 (0.44, 2.52)	244	1.05 (0.42, 2.61)
Nonmovers
≥ 6 hr/day	329	1.40 (0.63, 3.10)	294	1.21 (0.52, 2.82)	291	1.33 (0.56, 3.13)
< 6 hr/day	193	1.31 (0.50, 3.44)	172	1.08 (0.38, 3.07)	166	1.11 (0.38, 3.27)
BMI
BMI (> 26.5 kg/m^2^)
All men	617	2.01 (1.13, 3.60)	563	1.70 (0.92, 3.14)	554	1.76 (0.94, 3.28)
Most precise exposure^*e*^	510	2.16 (1.14, 4.09)	467	1.87 (0.95, 3.66)	459	2.01 (1.01, 3.98)
Nonmovers	425	2.38 (1.20, 4.73)	388	2.02 (0.98, 4.18)	383	2.03 (0.97, 4.24)
Nonmovers with most precise exposure^*e*^	388	2.65 (1.29, 5.45)	355	2.28 (1.06, 4.89)	351	2.35 (1.08, 5.10)
BMI (12–26.5 kg/m^2^)
All men	457	0.91 (0.48, 1.73)	410	1.06 (0.54, 2.11)	396	1.15 (0.57, 2.33)
Most precise exposure^*e*^	370	0.98 (0.48, 2.00)	338	1.15 (0.54, 2.44)	327	1.35 (0.62, 2.92)
Nonmovers	304	0.95 (0.43, 2.10)	282	1.08 (0.47, 2.47)	270	1.25 (0.54, 2.93)
Nonmovers with most precise exposure^*e*^	269	1.01 (0.44, 2.36)	251	1.16 (0.48, 2.78)	241	1.41 (0.58, 3.49)
^***a***^ICD-10 codes I10–I70. ^***b***^Age as follow-up time and adjusted for sex, state of enrollment, and birth year; ^***c***^Minimally adjusted plus covariates for pack-years of smoking and BMI; ^***d***^Moderately adjusted plus covariates for marital status, education level, alcoholic drinks per month, and vegetable intake. ^***e***^Excluding participants with PM_2.5_ exposure assigned to a ZIP code centroid.						

**Figure 2:**
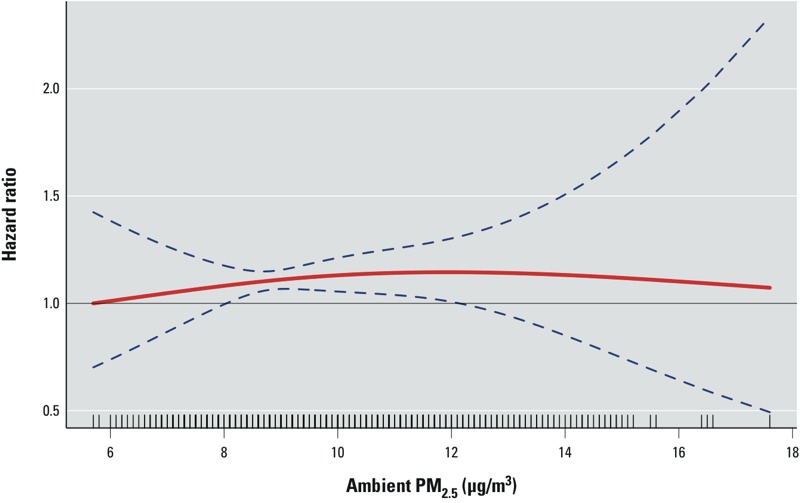
PM_2.5_ concentration–response curve (solid red line) and 95% CI (blue dashed lines) for cardiovascular mortality in men based on natural spline models with 2 degrees of freedom. The Cox model was stratified by state of enrollment and birth year category and adjusted for BMI (kg/m^2^), pack-years of smoking, marital status, education, alcohol consumption, and vegetable consumption.

Little change was noted in the PM_2.5_ coefficient for cardiovascular mortality in men when a shared frailty term for county was included in the minimally adjusted model (data not shown); therefore, excluding this term from the main analysis likely did not have a dramatic impact on model coefficients. Concentration–response plots for ambient PM_2.5_ and cardiovascular mortality in men suggested a linear increase in the risk of cardiovascular mortality at low PM_2.5_ concentrations (< 10 μg/m^3^) (Figure 2).

Ambient PM_2.5_ concentrations were not correlated with occupational exposures examined in the sensitivity analyses (*r* < 0.13), and additional adjustment for these factors had little impact on model estimates for PM_2.5_ (see Supplemental Material, Table S2). Similarly, including additional variables for smoking status at enrollment, cigarettes per day, years smoked, or the use of pipes, cigars, or chewing tobacco did not have a meaningful impact on coefficients for PM_2.5_ (see Supplemental Material, Table S2). Among men, adjusting for physical activity increased PM_2.5_ coefficients in the minimally adjusted model (HR = 1.30; 95% CI: 0.77, 2.19) but decreased coefficients in the moderately (HR = 1.08; 95% CI: 0.61, 1.91) and fully adjusted models (HR = 1.08; 95% CI: 0.60, 1.93).

As expected, BMI and pack-years of smoking were each important predictors of nonaccidental and cardiovascular mortality. Specifically, in moderately adjusted models each 10-unit increase in BMI was associated with a 17% increased risk of nonaccidental mortality (95% CI: 1.09, 1.26), whereas a 10-unit increase in pack-years of smoking was associated with a 12% increased risk of nonaccidental mortality (95% CI: 1.01, 1.14). Similar increases in BMI and pack-years of smoking were associated with 29% (95% CI: 1.13, 1.47) and 11% (95% CI: 1.08, 1.13) increased risks of cardiovascular mortality, respectively. Sensitivity analyses conducted with missing data for BMI and vegetable intake replaced with sex-specific mean values produced estimates similar to those of the minimally adjusted models; however, ambient PM_2.5_ was not associated with a significant decrease in nonaccidental mortality among women in this analysis (see Supplemental Material, Tables S3).

When models for cardiovascular mortality were stratified by BMI, HRs were consistently higher among men in the highest category of BMI (> 26.5 kg/m^2^) (Table 3). For example, the HR in the fully adjusted model was 1.76 (95% CI: 0.94, 3.28) among men in the highest category of BMI compared with 1.15 (95% CI: 0.57, 2.33) in the low BMI category. When limited to men with the most precise exposure assignment (i.e., excluding men with exposure assigned to a ZIP code centroid), the HR for cardiovascular mortality increased to 2.01 (95% CI: 1.01, 3.98) for men in the highest category of BMI relative to 1.35 (95% CI: 0.62, 2.92) in the low BMI category. Although these findings suggest potential effect modification by BMI, we had limited power to detect such effects, and tests for interaction between PM_2.5_ and BMI were not statistically significant (*p* > 0.05).

Of the occupational exposures examined, only diesel tractor use was independently associated with mortality in the AHS cohort. Specifically, diesel tractor use was associated with nonaccidental mortality among men in minimally (HR = 1.11; 95% CI: 1.01, 1.23), moderately (HR = 1.19; 95% CI: 1.07, 1.33), and fully adjusted models (HR = 1.23; 95% CI: 1.10, 1.37). Diesel tractor use was also associated with increased risks of cardiovascular mortality in men in minimally (HR = 1.08; 95% CI: 0.92, 1.27), moderately (HR = 1.16; 95% CI: 0.97, 1.39), and fully adjusted models (HR = 1.21; 95% CI: 1.00, 1.45). However, this analysis was limited to men because few women reported diesel tractor use (< 300). The magnitude of association between PM_2.5_ and cardiovascular mortality among men was similar among users and non-users of diesel tractors (data not shown).

## Discussion

Few cohort studies have examined the long-term health impacts of PM_2.5_ in rural areas. This is an important question because there may be systematic differences in particle composition between urban and rural regions (Aneja et al. 2006; Edgerton et al. 2005). In this study, ambient PM_2.5_ was not associated with nonaccidental mortality in the AHS cohort as a whole, but inverse relationships were observed among women. Conversely, consistent positive associations were observed between ambient PM_2.5_ and cardiovascular mortality in men, with the strongest associations observed among men who did not change their enrollment address. Although not statistically significant, the magnitude of association between PM_2.5_ and cardiovascular mortality among men (8%–43% increase per 10-μg/m^3^ increment) was similar to values reported in previous studies of PM_2.5_ and cardiovascular mortality (Hoek et al. 2013). Specifically, each 10-μg/m^3^ increment in ambient PM_2.5_ was associated with 26–28% increases in cardiovascular mortality in the Harvard Six Cities Study (Laden et al. 2006; Lepeule et al. 2012), a 12% increase in the American Cancer Society Cohort (Pope et al. 2004), and a 16% increase in the Canadian Census Cohort (Crouse et al. 2012); however, in these studies increases were not limited to men, and inverse associations were not reported among women. In addition, our analysis limited to nonmoving men with the most precise exposure geocoding resulted in risk estimates that were higher than in previous studies. Nonetheless, two of these previous studies (Crouse et al. 2012; Lepeule et al. 2012) noted linear concentration–response relationships for cardiovascular mortality at low PM_2.5_ concentrations, and our findings are consistent with these results. Moreover, these findings are of particular interest because they suggest increased risks of cardiovascular mortality for both rural and urban PM_2.5_ concentrations below most national and international standards. More generally, our findings suggest that long-term exposure to ambient PM_2.5_ may have adverse health effects among healthy populations similar to those included in the AHS (Waggoner et al. 2011). However, at least one other study in an occupational cohort of men did not observe a positive association between cardiovascular mortality and ambient PM_2.5_ (Puett et al. 2011). Nevertheless, this previous study (Puett et al. 2011) was conducted among a population of male health professionals (e.g., dentists, pharmacists, optometrists) working indoors away from home, so the findings may not be directly comparable to those for men in the AHS. In particular, exposures assigned to addresses for male health professionals may not reflect personal exposures as well as those for men in the AHS cohort, who work predominantly outdoors close to home.

In contrast to previous evidence suggesting a strong relationship between ambient PM_2.5_ and cardiovascular mortality in women (Chen et al. 2005; Miller et al. 2007; Ostro et al. 2010; Puett et al. 2009), a similar association was not observed in the AHS cohort. In particular, statistically significant inverse relationships were observed between ambient PM_2.5_ and nonaccidental mortality among women; however, this finding disappeared in several sensitivity analyses. On the other hand, Miller et al. (2007) and Puett et al. (2009) each reported stronger PM_2.5_ effect estimates for cardiovascular morbidity/mortality among subjects with higher BMIs and our findings among men are consistent with these results. Nonetheless, reasons for other discrepancies are not clear. One explanation may be differences in particle composition between rural and urban areas, although this seems unlikely given the positive association between ambient PM_2.5_ and cardiovascular mortality among men. If occupational PM_2.5_ exposures (or some other occupational exposure associated with cardiovascular mortality) among men were correlated with ambient concentrations, this may explain the discrepancy between men and women with respect to cardiovascular mortality; however, none of the occupational activities we examined were associated with ambient PM_2.5_. Alternatively, this difference may be explained in part by increased exposure measurement error for women in the AHS because they reported spending much less time outdoors during the growing season relative to men. In addition, many women in the AHS worked away from the home. Unfortunately, we do not have detailed information on the types and locations (i.e., indoors/outdoors) of jobs held by women in the AHS during follow-up, and thus cannot evaluate this question. In general, it is unclear which unmeasured exposures/risk factors may potentially explain the inverse associations noted between ambient PM_2.5_ and nonaccidental/cardiovascular mortality among women.

Although this study has many advantages including detailed individual-level covariate data for known cardiovascular disease risk factors and PM_2.5_ exposure assignment over a large rural area, it is important to recognize several limitations. First, the findings are likely not generalizable to all rural residents given the occupational nature of the cohort. In addition, as in all epidemiological studies, we cannot rule out the possibility of residual confounding; however, associations between PM_2.5_ and cardiovascular mortality in men were robust to adjustment for a number of individual-level covariates.

For occupational exposures specifically, our adjustment was limited to broad (ever/never) classifications of job activities such as welding or repairing engines that may not adequately capture occupational exposures to PM_2.5_ or other exposures such as coarse PM. However, to explain the observed positive association with cardiovascular mortality in men, occupational exposures would have to be strongly correlated with ambient concentrations, and we observed only weak correlations between ambient PM_2.5_ and the job activities examined. In addition, any occupational activities that contribute to personal exposures through recurring ambient emissions should be at least partially captured by remote sensing data. In general, the availability of even limited occupational data was an important advantage of this study as most large-scale studies of ambient air pollution and cardiovascular mortality lack individual-level data on occupational exposures/activities. Furthermore, the finding of a positive association between diesel tractor use and nonaccidental mortality after adjusting for individual-level risk factors is of particular interest and warrants further evaluation because diesel exhaust is classified as a human carcinogen (Benbrahim-Tallaa et al. 2012).

Although our surrogate measure of time spent outdoors (i.e., time in the sun) was likely an imperfect measure of total time exposed to outdoor air, the ability to examine mortality risk by estimated time outdoors is an important advantage. However, our definition of time outdoors may miss those individuals who work outdoors but avoid the sun (e.g., working in tractors with enclosed cabins).

Exposure misclassification also remains a concern because PM_2.5_ concentrations assigned to participants’ residences reflected average values between 2001 and 2006 and thus only a portion of the follow-up time. However, this time period captures several years approximately centered across the entire follow-up period (1993–2011), and thus seems like a reasonable estimate of average ambient concentrations during this time. A further limitation is that we did not have information on the amount of time participants spent at home during the follow-up period, and we did not have time-varying exposure information for participants that moved. As a result, exposures assigned to residences may have under- or overestimated true annual average concentrations for the duration of follow-up. However, this misclassification was likely nondifferential, and thus is not likely to explain the observed positive association between PM_2.5_ and cardiovascular mortality in men. Likewise, use of ZIP code centroids for geocoding for a portion of participants likely had a similar effect. However, because farmers generally work outdoors in close proximity to their home, this misclassification may be smaller than in previous cohort studies. In addition, sensitivity analyses aimed at refining exposure estimates resulted in stronger associations between PM_2.5_ and cardiovascular mortality in men, thus suggesting that exposure measurement error may have biased risk estimates toward the null.

Another limitation is the relatively low spatial resolution of PM_2.5_ exposure estimates, which limited our ability to resolve small spatial differences in air pollutant concentrations. However, this may be less of a concern in rural areas than in urban locations, which likely have a larger number of PM_2.5_ sources in a given 10 × 10 km area (and thus more spatial variation). In addition, data for several individual-level covariates examined in sensitivity analyses were limited to a subset of the entire cohort, and as a result risk estimates for these models were less precise, owing to decreased sample size; however, these factors did not appear to have an important impact on model coefficients for PM_2.5_.

## Conclusion

In general, our findings suggest that rural PM_2.5_ may contribute to cardiovascular mortality in men. However, long-term exposure to PM_2.5_ was not associated with a significantly increased risk of cardiovascular mortality for the cohort as a whole, and no evidence of increased risk was observed among women. Further evaluation is needed to clarify reasons for sex differences in the relationship between ambient PM_2.5_ and mortality in the AHS cohort.

## Supplemental Material

(468 KB) PDF
