# Supplementary material for: Long-Term Exposure to Fine Particulate Matter: Association with Nonaccidental and Cardiovascular Mortality in the Agricultural Health Study Cohort
Source: Environ Health Perspect. 2014 Mar 14;122(6):609–15. doi: 10.1289/ehp.1307277 (PMC4050514; doi:10.1289/ehp.1307277)

Environmental Health Perspectives doi:10.1289/ehp.1307277

Supplemental Material

Long-Term Exposure to Fine Particulate Matter: Association with Nonaccidental and Cardiovascular Mortality in the Agricultural Health Study Cohort

Scott Weichenthal, Paul J. Villeneuve, Richard T. Burnett, Aaron van Donkelaar, Randall V. Martin, Rena R. Jones, Curt T. DellaValle, Dale P. Sandler, Mary H. Ward, and Jane A. Hoppin

Table of Contents **Page**

Table S1. Hazard ratios for nonaccidental mortality per 10 μg/m^3 increase in ambient $PM_{2.5}$ among participants in the Agricultural Health Study, 1993-2011 (excluding participants with exposures assigned to Zip-code centroids) 2

Table S2. Hazard ratios for cardiovascular mortality per 10 μg/m^3 increase in ambient $PM_{2.5}$ among men in the Agricultural Health Study, 1993-2011 with adjustment for additional smoking variables and occupational exposures 3

Table S3. Hazard ratios for nonaccidental and cardiovascular mortality per 10 μg/m^3 increase in ambient $PM_{2.5}$ among participants in the Agricultural Health Study, 1993-2011 (missing BMI and vegetable intake values replaced with sex-specific mean values) 4

Figure S1. Correlation between remote-sensed $PM_{2.5}$ and surface concentrations in Iowa and North Carolina between 2001-2006. Crosses on each map indicate monitoring locations 5

Table S1. Hazard ratios for nonaccidental mortality[a] per 10 μg/m^3 increase in ambient $PM_{2.5}$ among participants in the Agricultural Health Study, 1993-2011 (excluding participants with exposures assigned to Zip-code centroids)

Population	No. Deaths	Minimally Adjusted[b] HR (95% CI)	No. Deaths	Moderately Adjusted[c] HR (95% CI)	No. Deaths	Fully Adjusted[d] HR (95% CI)
All	4774	0.96 (0.78, 1.18)	3353	0.98 (0.77, 1.26)	3268	1.02 (0.79, 1.31)
Men	3409	0.98 (0.77, 1.26)	2289	1.06 (0.79, 1.43)	2226	1.15 (0.85, 1.56)
Women	1365	0.90 (0.60, 1.34)	1064	0.81 (0.51, 1.29)	1042	0.79 (0.49, 1.26)
Non-Movers						
All	3538	0.94 (0.74, 1.20)	2441	0.89 (0.67, 1.20)	2375	0.95 (0.70, 1.28)
Men	2500	1.00 (0.75, 1.32)	1642	1.04 (0.74, 1.48)	1593	1.15 (0.80, 1.65)
Women	1038	0.82 (0.52, 1.29)	799	0.62 (0.36, 1.06)	782	0.62 (0.36, 1.07)

Abbreviations: CI, confidence interval; HR, hazard ratio. [a]ICD-10 codes I10-I70; [b]Age as follow-up time and adjusted for gender, State of enrollment, and birth year; [c]Minimally adjusted plus covariates for pack-years of smoking and body mass index; [d]Moderately adjusted plus covariates for marital status, education level, alcoholic drinks per month, and vegetable intake.

Table S2. Hazard ratios for cardiovascular mortality[a] per 10 μg/m^3 increase in ambient $PM_{2.5}$ among men in the Agricultural Health Study, 1993-2011 with adjustment for additional smoking variables and occupational exposures.

**Additional Covariates**	**No. Deaths**	**Minimally Adjusted[b] HR (95% CI)**	**No. Deaths**	**Moderately Adjusted[c] HR (95% CI)**	**No. Deaths**	**Fully Adjusted[d] HR (95% CI)**
Primary model	1534	1.08 (0.75, 1.56)	973	1.37 (0.87, 2.17)	950	1.43 (0.89, 2.27)
**Plus (separately):**						
Occupational Exposures						
Repair Engines	1458	1.08 (0.75, 1.57)	934	1.40 (0.88, 2.24)	911	1.46 (0.91, 2.35)
Weld	1526	1.13 (0.78, 1.63)	966	1.42 (0.90, 2.24)	943	1.48 (0.92, 2.36)
Years Mix/ Apply Pesticide	1378	1.09 (0.74, 1.61)	923	1.40 (0.88, 2.25)	902	1.47 (0.91, 2.39)
Grind metal	1534	1.09 (0.75, 1.56)	973	1.38 (0.88, 2.18)	950	1.44 (0.90, 2.29)
Diesel Tractors	1534	1.09 (0.75, 1.56)	973	1.38 (0.87, 2.17)	950	1.44 (0.90, 2.29)
Gas Tractors	1534	1.07 (0.74, 1.54)	973	1.38 (0.87, 2.17)	950	1.43 (0.89, 2.28)
Physical Activity	742	1.30 (0.77, 2.19)	651	1.08 (0.61, 1.91)	639	1.08 (0.60, 1.93)
Smoking						
Current/former/never	1447	1.08 (0.74, 1.58)	971	1.35 (0.86, 2.13)	948	1.40 (0.88, 2.23)
Pipe	1526	1.10 (0.76, 1.58)	966	1.39 (0.88, 2.20)	943	1.45 (0.91, 2.32)
Cigars	1526	1.10 (0.76, 1.59)	966	1.40 (0.88, 2.22)	943	1.45 (0.91, 2.32)
Chew Tobacco	1526	1.10 (0.76, 1.58)	966	1.39 (0.88, 2.20)	943	1.45 (0.90, 2.31)
Cigs/day	1405	1.07 (0.73, 1.56)	973	1.38 (0.87, 2.17)	950	1.43 (0.89, 2.27)
Years smoked	1416	1.13 (0.77, 1.66)	973	1.38 (0.87, 2.18)	950	1.43 (0.90, 2.28)

Abbreviations: CI, confidence interval; HR, hazard ratio. [a]ICD-10 codes I10-I70; [b]Age as follow-up time and adjusted for gender, State of enrollment, and birth year; [c]Minimally adjusted plus covariates for pack-years of smoking and body mass index; [d]Moderately adjusted plus covariates for marital status, education level, alcoholic drinks per month, and vegetable intake.

Table S3. Hazard ratios for nonaccidental and cardiovascular mortality per 10 μg/m^3 increase in ambient $PM_{2.5}$ among participants in the Agricultural Health Study, 1993-2011 (missing BMI and vegetable intake values replaced with sex-specific mean values).

Cause of Death	No. Deaths	Minimally Adjusted[a] HR (95% CI)	No. Deaths	Moderately Adjusted[b] HR (95% CI)	No. Deaths	Fully Adjusted[c] HR (95% CI)
**Non-Accidental[d]**						
All	5929	0.92 (0.76, 1.11)	5397	0.93 (0.76, 1.13)	5243	0.94 (0.77, 1.15)
Men	4271	0.97 (0.78, 1.20)	3891	0.96 (0.76, 1.21)	3770	1.00 (0.79, 1.27)
Women	1658	0.80 (0.56, 1.15)	1506	0.85 (0.58, 1.25)	1473	0.82 (0.55, 1.21)
Non-Movers: All	4019	0.85 (0.68, 1.06)	3661	0.86 (0.68, 1.09)	3554	0.90 (0.70, 1.15)
Non-Movers: Men	2860	0.93 (0.71, 1.21)	2611	0.94 (0.71, 1.25)	2527	1.02 (0.76, 1.36)
Non-Movers: Women	1159	0.67 (0.44, 1.03)	1050	0.67 (0.43, 1.06)	1027	0.66 (0.41, 1.05)
**Cardiovascular[e]**						
All	1967	1.00 (0.72, 1.37)	1757	1.04 (0.73, 1.47)	1704	1.02 (0.71, 1.45)
Men	1534	1.08 (0.75, 1.56)	1378	1.08 (0.73, 1.59)	1336	1.07 (0.72, 1.59)
Women	433	0.73 (0.37, 1.46)	379	0.91 (0.43, 1.93)	368	0.84 (0.39, 1.81)
Non-Movers: All	1357	1.00 (0.67, 1.46)	1220	1.02 (0.68, 1.55)	1184	1.06 (0.70, 1.62)
Non-Movers: Men	1043	1.16 (0.75, 1.80)	946	1.16 (0.73, 1.85)	917	1.21 (0.75, 1.95)
Non-Movers: Women	314	0.59 (0.26, 1.32)	274	0.66 (0.27, 1.61)	267	0.64 (0.26, 1.60)

Abbreviations: CI, confidence interval; HR, hazard ratio. [a]Age as follow-up time and adjusted for gender, State of enrollment, and birth year; [b]Minimally adjusted plus covariates for pack-years of smoking and body mass index; [c] Moderately adjusted plus covariates for marital status, education level, alcoholic drinks per month, and vegetable intake.[d] ICD-10 codes <V01; [e]ICD-10 codes I10-I70.

Figure S1. Correlation between remote-sensed $PM_{2.5}$ and surface concentrations in Iowa and North Carolina between 2001-2006. Crosses on each map indicate monitoring locations.

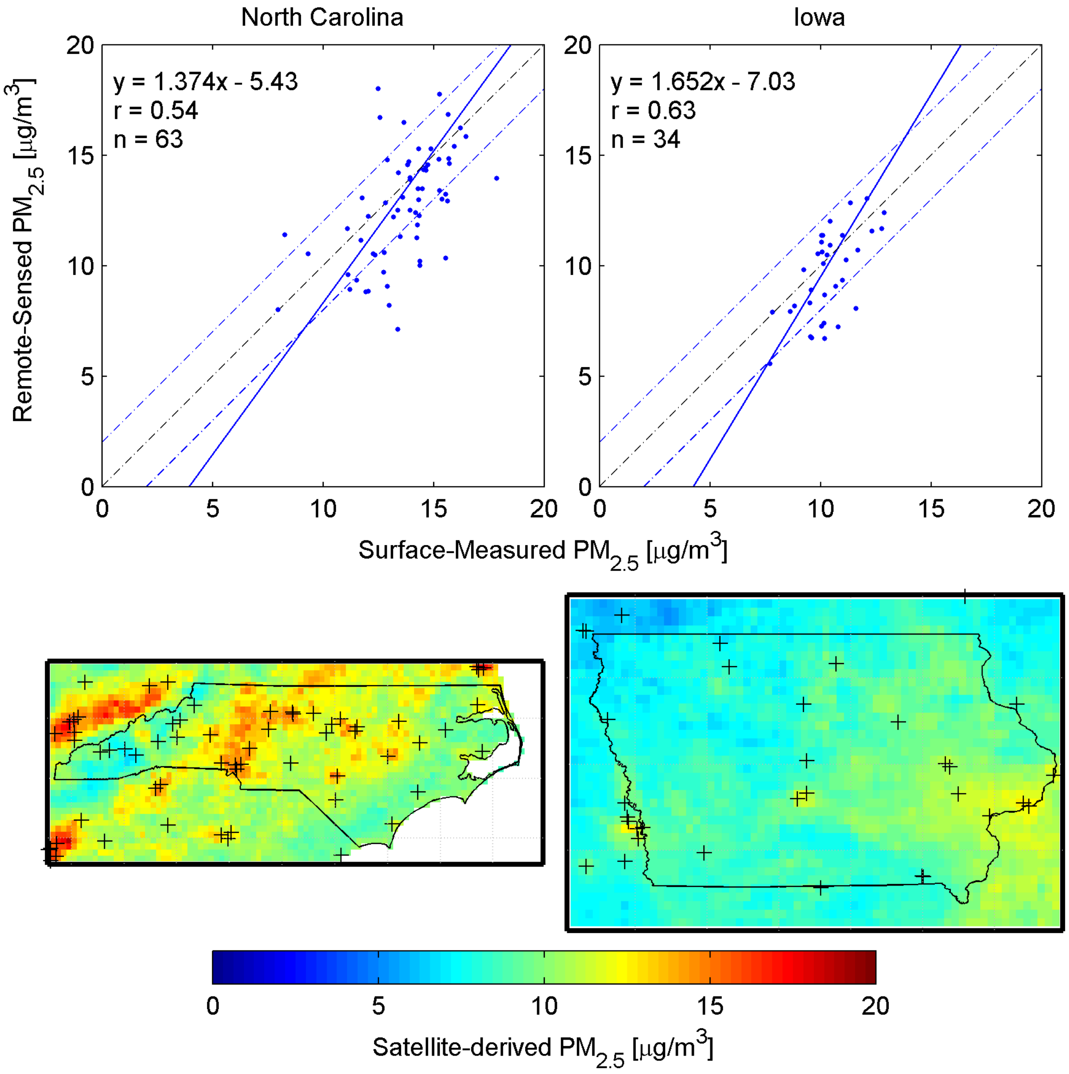

Supplement: (468 KB) PDF [file ehp.1307277.s001.pdf]
